# Network analysis of microRNAs, genes and their regulation in diffuse and follicular B-cell lymphomas

**DOI:** 10.18632/oncotarget.23974

**Published:** 2018-01-05

**Authors:** Oshrat Hershkovitz-Rokah, Polina Geva, Mali Salmon-Divon, Ofer Shpilberg, Stella Liberman-Aronov

**Affiliations:** ^1^ Department of Molecular Biology, Faculty of Natural Sciences, Ariel University, Ariel, Israel; ^2^ Translational Research Laboratory, Assuta Medical Centers, Tel Aviv, Israel; ^3^ Institude of Hematology, Assuta Medical Centers, Tel Aviv, Israel; ^4^ Pre-Medicine Department, School of Health Sciences, Ariel University, Ariel, Israel

**Keywords:** miRNA signature, DLBCL, FL, miRNA-mRNA pairing, B cell lymphoma

## Abstract

MicroRNAs (miRs) are short non-coding regulatory RNAs that control gene expression at the post-transcriptional level and play an important role in cancer development and progression, acting either as oncogenes or as tumor suppressors. Identification of aberrantly expressed miRs in patients with hematological malignancies as compared to healthy individuals has suggested that these molecules may serve as novel clinical diagnostic and prognostic biomarkers.

We conducted a systematic literature review of articles published between 2007 and 2017 and re-analyzed experimentally-validated human miR expression signatures in diffuse large B-cell lymphoma (DLBCL) and follicular lymphoma (FL) from various biological sources (tumor tissue, peripheral blood, bone marrow and cell lines). A unique miR expression pattern was observed for each disease. Compared to healthy individuals, 61 miRs were aberrantly expressed in DLBCL and 85 in FL; 20-30% of aberrantly expressed miRs overlapped between the two lymphoma subtypes.

Analysis of integrative positive and negative miRNA-mRNA relationships using the Ingenuity Pathway Analysis (IPA) system revealed 970 miR-mRNA pairs for DLBCL and 90 for FL. Through gene ontology analysis, we found potential regulatory pathways that are deregulated in DLBCL and FL due to improper expression of miR target genes. By comparing the expression level of the aberrantly expressed miRs in DLBCL to their expression levels in other malignancies, we identified seven miRs that are aberrantly expressed in DLBCL tumor tissues (miR-15a, miR-16, miR-17, miR-106, miR-21, miR-155 and miR-34a-5p). This specific expression pattern may be a potential diagnostic tool for DLBCL.

## INTRODUCTION

Diffuse Large B-cell Lymphoma (DLBCL) and Follicular B-cell Lymphoma (FL) are the most common subtypes of non-Hodgkin lymphoma (NHL) [1]. FL is the most common indolent form, accounting for approximately 20% to 30% of all NHLs [2]. FL can become resistant to most conventional chemotherapies and may transform into the more aggressive DLBCL [2], which is the most common fast-growing NHL. DLBCL shows clinical, pathological and molecular heterogeneities. Presumably due to this molecular heterogeneity, about 30% to 40% of DLBCL patients do not respond well to common therapy regimens [2–4]. Recent advances in gene expression profiling and genome-sequencing analyses have led to the identification of distinct molecular DLBCL and FL subtypes that are distinguished by altered gene expression, DNA mutations or chromosomal translocation.

MicroRNAs (miRs) are short (19–24 nucleotides) non-coding RNAs that affect the regulation of gene expression by binding to the 3′-untranslated region (3′ UTR) within target messenger RNAs (mRNAs). MiRs regulate critical cell processes such as metabolism, apoptosis, development and cell-cycle [5, 6]. MiRs are very stable in most types of tissues and in extracellular fluids such as plasma, serum, saliva, and urine [7, 8].

Precise regulation by miRs is essential for proper lineage decision during the development of the hematopoietic system [9] while its disruption leads to malignant transformation [10]. Many cancers, including hematological malignancies, display aberrant miR levels. Similar patterns of expression were observed in tissue, cell-free, and circulating tumor cell-associated miRs [11]. These observations suggest that circulating miRs may serve as useful biomarkers for the diagnosis and monitoring of disease progression [12]. However, published miR expression profile data are variable and require further examination in order to improve the accuracy of lymphoma diagnosis and subsequent therapy selection.

In the present study, we aimed to identify, through a literature search and bioinformatics analysis, specific miR expression profiles of DLBCL and FL and to compare between them. In addition, we performed an integrative analysis of the identified miRs and their mRNA targets in order to find functional associations amongst them.

## RESULTS AND DISCUSSION

### Study identification

**Figure 1:**
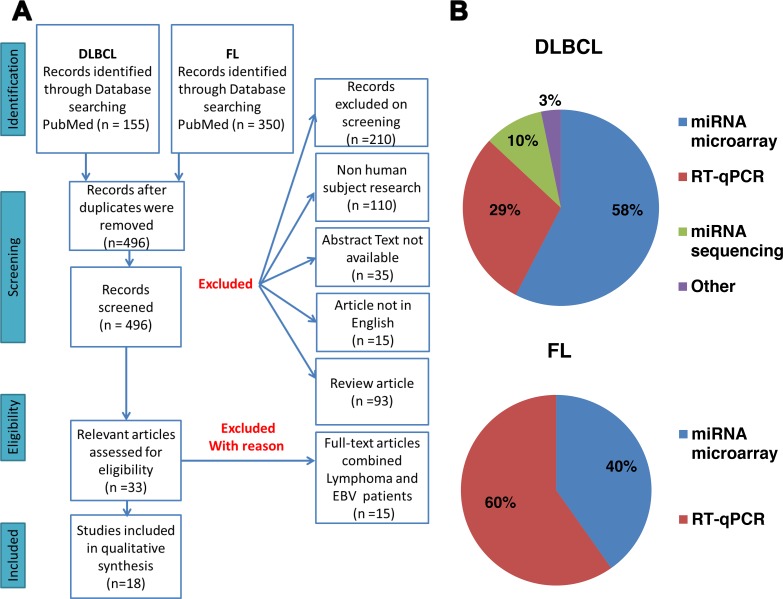
**(A)** Flow diagram of the article selection process. **(B)** Main molecular techniques used for miR detection in the literature. DLBCL = diffuse large B-cell lymphoma; FL = follicular lymphoma.

Our systematic search identified 155 studies on DLBCL and 350 studies on FL that were published between 2007 and 2017 and screened according to the flowchart presented in Figure 1A. Overall, 18 articles were included in the final analysis, ten papers on DLBCL, four papers on FL, and four that were common to both diseases. MiRs that were significantly up- or downregulated in DLBCL or FL patients as compared to healthy individuals were selected for further analysis.

### Analysis of the publications retrieved in the literature search

#### Molecular techniques of miRNA detection

Our analysis of the publications that were retrieved in the literature search showed that the most common methods for identifying miR expression signature in samples from patients were miR microarray (58% for DLBCL and 40% for FL) and real-time quantitative polymerase chain reaction (RT-qPCR) (29% for DLBCL and 60% for FL), (Figure 1B). These techniques are widely used and suitable for accurately quantifying miRs. Other methods for miR identification included miR sequencing and invader miRNA assays. The majority of miRs (69%) were further validated by a second method, the most common of which was RT-qPCR.

#### Mapping of identified miRs in DLBCL and FL

Our literature search revealed 61 aberrantly expressed miRs in DLBCL and 84 in FL, as compared to healthy controls. High variability of miR expression levels and contradictory results were observed among some of the studies. In order to increase the accuracy of our findings, we collected only 20 of the most differentially expressed miRs as defined by the authors of the selected studies for further analysis as potential biomarkers of DLBCL and FL (Supplementary Tables 1 and 2).

**Figure 2:**
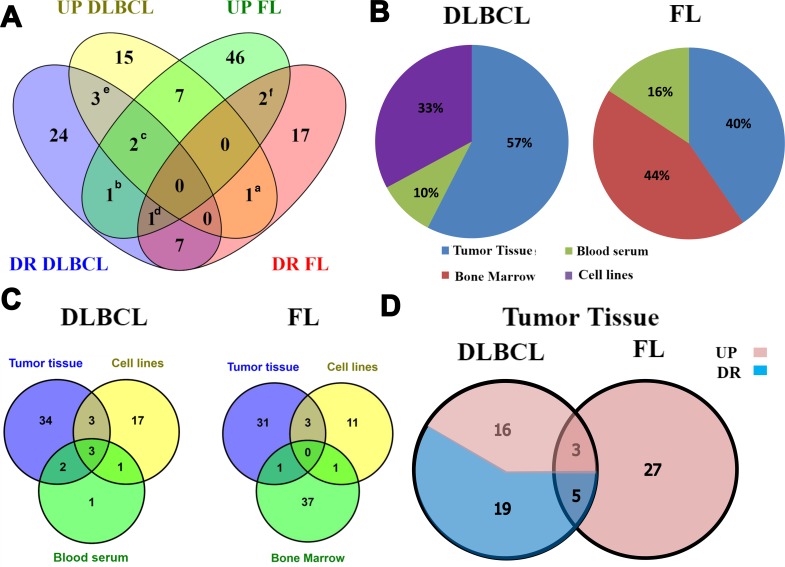
Experimentally validated MicroRNAs (miRs) found in diffuse large B-cell lymphoma (DLBCL) and Follicular lymphoma (FL) retrieved in the literature search **(A)** A Venn diagram representing overlapping up- and downregulated miRs in DLBCL and FL. (a) miR-494 is upregulated in DLBCL and downregulated in FL; (b) miR-181a is downregulated in DLBCL and upregulated in FL; (c) miR-16 and miR-19b that are up- and downregulated in DLBCL and upregulated in FL; (d) miR-29a is up- and downregulated in FL and downregulated in DLBCL; (e) miR-17-5p, miR-145-5p and miR-15a are up- and downregulated in DLBCL; (f) miR-17-3p and miR-202 are up- and downregulated in FL. **(B)** The percentage of aberrantly-expressed miRs in each biological source of DLBCL and FL. No evidence of miR expression data in the bone marrow of DLBCL patients and in the blood serum in FL patients were found. **(C)** A Venn diagram representing the number of overlapping miRs among biological sources. **(D)** A Venn diagram representing the number of overlapping up- and downregulated miRs in tumor tissue from DLBCL (n=43) and FL (n=35). ^*^miR-145-5p was counted twice in DLBCL according to evidence of up- and downregulation.

To that end, we compared the miR expression profile of DLBCL and FL. We observed a distinct pattern of abnormally expressed miRs in these lymphoma subtypes: 15 unique miRs were upregulated and 24 were downregulated in DLBCL. In FL, 46 unique miRs were upregulated and 17 were downregulated. In addition, DLBCL and FL had seven common upregulated miRs and seven common downregulated miRs (Figure 2A and Supplementary Tables 1 and 2). MiRs identified in this analysis include miR-320, 34a, 155, 21 and miR-210, which have been previously reported as potential biomarkers in other cancers such as osteosarcoma, lung cancer, breast cancer, myeloid leukemia, high-grade glioma, colon cancer and hepatoma [13–19]. MiR-494 was upregulated in DLBCL and downregulated in FL, while miR-181a showed an opposite expression pattern, suggesting that these miRs may serve as potential specific biomarkers for both DLBCL and FL.

Several other miRs considered as biomarkers for DLBCL (miR-17-5p, 145-5p and miR-15a, Figure 2A, 2e) and FL (miR-17-3p and miR-202, Figure 2A, 2f) showed opposite expression levels in different biological sources. For example, in patients with DLBCL miR-15a was upregulated in blood serum [20] and downregulated in tumor lymph nodes [21] possibly due to distinct regulatory mechanisms. MiRs-16, 19b and miR-29a (Figure 2A, 2C-2D), were also detected in both lymphoma subtypes either with low or high expression levels.

To better characterize the types of miRs identified in various biological tissues in each lymphoma subtype and to define unique tissue-specific miRs, we compared their reported distribution in biological samples (Figure 2B and 2C). In DLBCL, most of the aberrantly expressed miRs (42 miRs, 57%) were purified from tumor tissue (lymph nodes). Twenty-four miRs (33%) were purified from cell lines, and only seven miRs (10%) were purified from peripheral blood (miRs-155, −21, 15a, 210 34a-5p, 16-1 and miR-29c). Of them, six were also found in lymph node tissue and cell lines (Figure 2C and Supplementary Table 1). Only miR-29c is uniquely overexpressed in blood. The miR-29 family is downregulated in peripheral blood of mantle cell lymphoma (MCL) patients [22] and may be used as a diagnostic and prognostic biomarker for MCL. Patients with significant downregulated miR-29 had shorter survival compared with those that expressed relatively high levels of miR-29c. Notably, miR-29c is expressed differently in MCL as compared to DLBCL and FL.

In FL, the most aberrantly expressed miRs were purified from tumor tissues. These included 35 miRs (40%) - 31 of which were specific - that were purified from lymph node tumor tissues during early stages (I-III) of the disease, and 39 miRs (44%) - 37 of which were specific - from bone marrow smears containing bone marrow tissue infiltrated by lymphoma cells at a more progressive stage (IV) of the disease [23]. Fifteen miRs (16%) were purified from cell lines (11 of which were specific).

Only two aberrantly expressed miRs (miR-17-3p and miR-202) overlapped among biological sources. Eleven unique miRs were aberrantly expressed in cell lines. The low number of overlapping miRs observed among biological sources of DLBCL and FL (Figure 2C) suggests that different tissues and biological sources have different regulatory networks that generate a particular set of expressed miRs. Moreover, the low number of overlapping miRs among cell lines and tumor tissues indicates that cell lines may not be the most useful source for assessing miRs for diagnostic purposes, probably because cell lines lack microenvironmental factors. Consequently, cell line models may be used mainly for understanding the molecular mechanism of the regulation of miRs-mRNA interactions.

Since most of the screened miRs were identified in tumor tissue, we further analyzed overlapping aberrantly expressed miRs that were detected in tumor tissue of DLBCL and FL patients. We found 16 up- and 19 downregulated unique miRs in DLBCL tumor tissue and 27 unique upregulated miRs in FL tumor tissue. To our surprise, no unique, downregulated miRs were observed in FL tumor tissue (Figure 2D and Supplementary Table 2), suggesting that a large number of genes regulated by these miRs are probably downregulated in the disease. The corresponding gene expression profiles were reported in several publications in the past [24, 25]. Altogether, both lymphoma subtypes had only eight common miRs: three that were upregulated (miR-210, 155 and miR-106a), and five that were downregulated (miR-139, 150, 149, 320 and miR-34a-5p) (Figure 2D). These results imply that each lymphoma subtype has a unique miR expression profile that may be used for differential diagnosis.

### Integrative analysis of miR and mRNA expression

To identify functional miR-mRNA relationships, we investigated associations between positive and negative miR-mRNA expression profiles. To that end, we utilized the GEO database (GSE12195) to examine inverse expression between the miRs retrieved by our literature search, and their targeted mRNAs using the Ingenuity Pathway Analysis (IPA) analysis. In DLBCL, 970 miR-mRNA pairs with opposite expression patterns were identified (Supplementary Tables 3A and 3B include data of miRs, genes and their generated pairs). Of 56 aberrantly expressed miRs identified in DLBCL, 45 showed mRNA-miR interactions using IPA.

**Figure 3:**
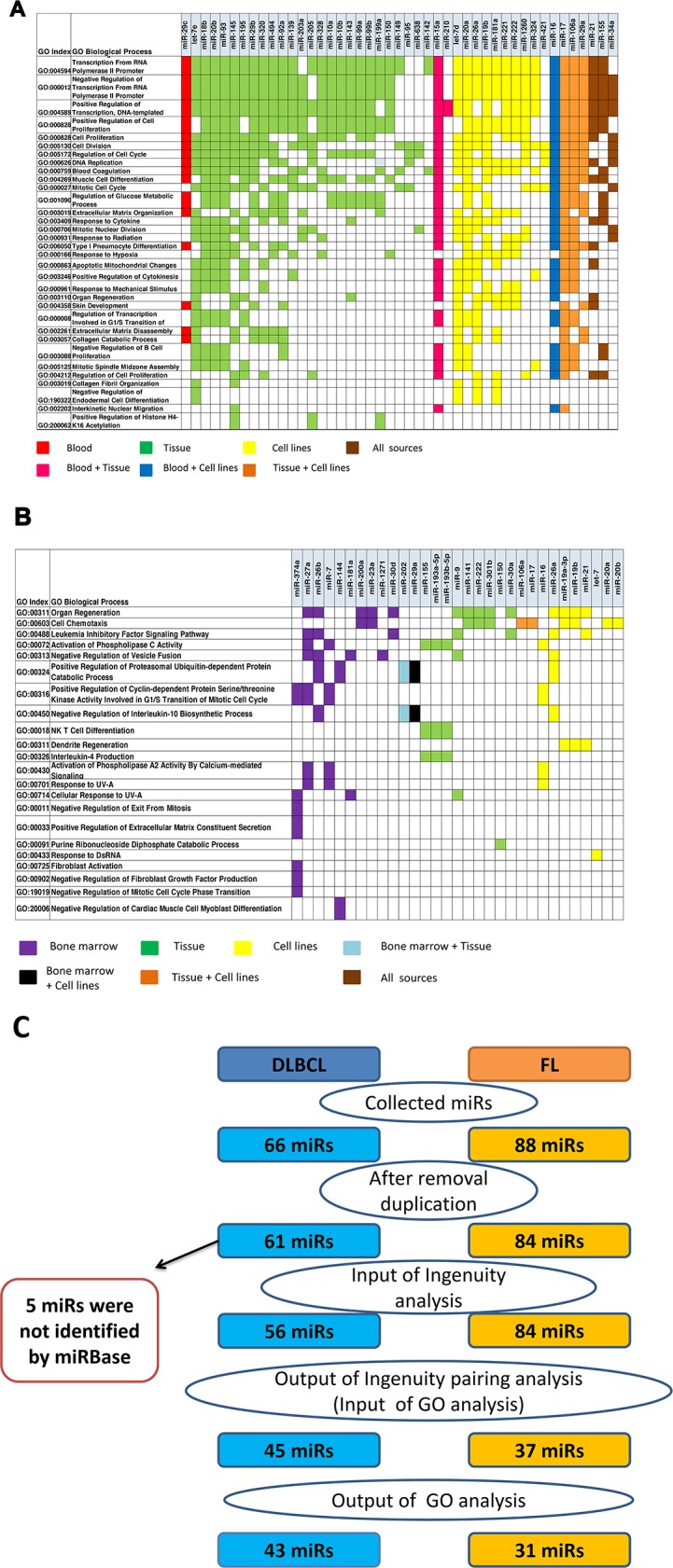
Ingenuity Pathways Analysis (IPA) pairing of miRs and gene groups that were significantly altered in DLBCL and FL were paired using gene ontology (GO) annotations associated with related miRNAs classified by their biological sources **(A)** GO analysis based on 970 miR-mRNA pairs (291 unique proteins) in DLBCL and **(B)** 90 miR-mRNA pairs (22 unique proteins) in FL were significantly enriched (p<0.01). **(C)** The steps and the number of miRs used for IPA and GO analyses. MiRs were removed from the analysis if they were duplicated or unrecognized by IPA or by miRbase version 20.

Next, we performed a gene ontology (GO) analysis to establish associations between miRs and the biological processes they regulate (Figures 3A-3B and Supplementary Tables 4A-4B). After GO enrichment analysis, 43 miRs remained, most of them identified in tumor tissues. Figure 3A presents the most statistically significant functional biological processes regulated by these miRs. The genes targeted by most of the miRs in Figure 3A are involved in the regulation of gene transcription and DNA replication; the most common processes relate to positive and negative transcription regulation of the RNA polymerase II promoter. Although it was initially suggested that most miR genes are transcribed by RNA Pol II [26], RNA Pol III-dependent transcription of miRs also occurs [27] and the transcriptional requirements of other miRs remains untested. In addition, some selected miRs regulate genes that are involved in cell cycle regulation, proliferation and division.

In FL, 90 miR-mRNA pairs with opposite expression patterns were identified using IPA. Only 31 out of 84 aberrantly expressed miRs showed significant involvement in specific biological processes. In contrast to DLBCL, it was difficult to find enriched biological processes for FL, possibly due to the small number (60) of down-expressed genes that were used for IPA compared to DLBCL, for which 845 downregulated genes were analyzed. In addition, because the regulatory role of miRs and their targets was less investigated in FL, fewer data were found in the literature and databases. MiRs that were over-expressed in FL, were associated with negative regulation of leukemia inhibitory factor signaling pathway, cell chemotaxis, organ regeneration, activation of phospholipase C activity, positive regulation of cyclin-dependent protein serine/threonine kinase activity involved in G1/S transition of the mitotic cell cycle, interleukin-10 biosynthetic process, natural killer T cell differentiation and Interleukin-4 production (Figure 3B). Although in both analyses – that of DLBCL and that of FL - we observed common biological processes such as cell proliferation and signal transduction pathways, the molecular basis of this regulation was different.

### Interaction network analyses in DLBCL

**Figure 4:**
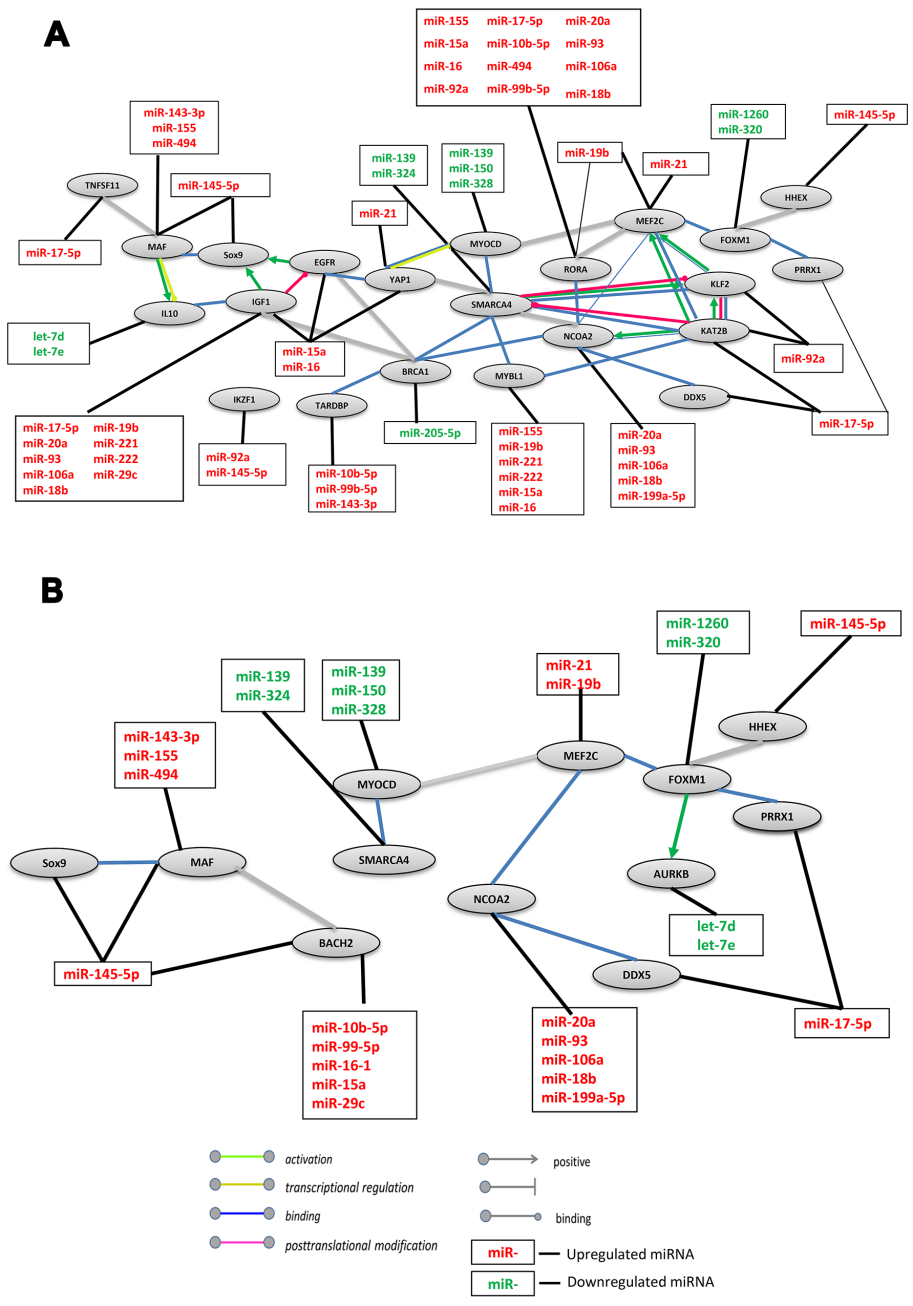
Network interaction between miRs and their target genes involved in RNA Polymerase II Promoter regulation in DLBCL **(A)** Positive regulation network. The gene group (GO:0045944) was taken from Gene Ontology annotations analysis. The STRING interaction database used high confidence interaction (0.7) between closed genes to create the network of targets in DLBCL. **(B)** Negative regulation network. The gene group (GO:0000122) was taken from Gene Ontology annotations analysis. The STRING interaction database used high confidence interaction (0.8) to generate the network targets in DLBCL. Up and downregulated updated miRs were added to the network.

To better understand the molecular mechanism of DLBCL malignancy regulation, we utilized the IPA tool to generate a miR-mRNA interaction network of positively-regulated transcription by the RNA Polymerase II promoter pathway (GO:0045944). Using the STRING software, we created a protein network of 39 functionally related genes (Supplementary Table 4A), whose mRNA expression level was altered in DLBCL, which were paired with 43 miRs retrieved in the literature search (Supplementary Table 1). The network showed a statistically significant strong interaction between the proteins with a high confidence score (>0.7), suggesting that these proteins are biologically related (Figure 4A). MiRs recorded in our previous analyses (Supplementary Table 3A) were manually added to the network. Although some interactions of miRs with their targets were expected based on previous studies, the network suggests a new mechanism that may be involved in the development of DLBCL.

Most of the proteins in this network are transcription factors that participate in various processes in several tissue types such as angiogenesis, DNA damage response, development, morphogenesis, differentiation and survival.

In the network A single gene may be regulated by several miRs (Figure 4A). For example, *IGF1* is regulated by nine miRs, *RORA* is regulated by twelve miRs and *MYBL1* is regulated by six miRs. Conversely, several genes may be regulated by a single miR. For example, miR-17-5p regulates four genes*, IGF1, RORA, KATB2* and *TNFSF11*. Hence, we suggest that this group of genes is commonly regulated. These findings may be explained by different temporary and spatial interactions among mRNAs and miRs. For example, the v-maf musculoaponeurotic fibro-sarcoma oncogene homolog (MAF) is a transcriptional activator or repressor that increases T-cell susceptibility to apoptosis by interacting with MYB and decreasing the expression of the anti-apoptotic protein BCL2 [28, 29]. According to our analysis *MAF* mRNA is downregulated by four miRs: miR-145-5p, 143-3p, 155 and 494, suggesting that if protein expression is reduced, BCL-2 gene expression is subsequently upregulated [30] leading to anti-apoptotic effects and contributing to the maintenance of abnormal cells and tumor development. This effect may also occur when one of the central network proteins, YAP1, is downregulated by miR-16. *YAP1* is a transcriptional regulator that plays a pivotal role in tumor suppression by inhibiting proliferation and promoting apoptosis [31].

Another interesting protein in this network is KAT2B, which interacts with five other proteins and activates two of them (MEF2C and NCOA2). This protein has histone acetyltransferase activity. It inhibits cell-cycle progression and counteracts the mitogenic activity of the adenoviral oncoprotein E1A [32]. We suggest that downregulation of *KATB* by miR-17-5p may disrupt cell cycle control, increasing neoplasm development.

Some of the proteins in this network are associated with B-cell development. For example, MYBL1, a v-myb myeloblastosis viral oncogene homolog, has a role in the proliferation and/or differentiation of B-lymphoid cells [33, 34]. As seen in Figure 4A, miRs-155, 19b, 221, 222, 15a and miR-16 downregulate its expression. Furthermore MYBL1 is a Myb-related protein A, whereas MYB is a transcriptional activator that acts in a dose-dependent manner in early B-lymphocyte development, stressing the importance of properly balanced regulatory networks in cell differentiation [35]. Therefore, downregulation of MYBL1 may disrupt B-cell differentiation.

*IKZF1* is regulated by miR-92a and miR-145-5p. It is one of the proteins associated with the GO process (GO:0045944) which was the basis of this network. Its relation to other proteins in the network has not yet been established, but because it is a key regulator of normal B-cell development [35], it may play a role in DLBCL development.

To complete our understanding regarding the role of miRs in the regulation of genes that participate in polymerase II promoter regulation, we built a miR-target network of negatively-regulated transcription of the RNA Polymerase II promoter that was based on the significant enrichment of genes belonging to this pathway (GO:0000122) in DLBCL (Figure 4B). For this analysis, we used 26 genes (Supplementary Table 4B), whose mRNAs are targeted by 38 miRs that were retrieved by our literature search (Supplementary Table 2) to build a strong-interaction network with a high confidence score (≥0.8). Twelve of the proteins also participate in positive regulation of the RNA Polymerase II promoter. The main protein complex used for this network (FOXM1-MEF2C-NCOA2-DDX5-MYOCD-SMARCA4) is conserved between DLBCL and FL. This group of transcription factors plays an important role in B cell maturation and proliferation. For example, FOXM1 is a master regulator of proliferation in the germinal center and is known as a human proto-oncogene. Upregulation of FOXM1 is involved in the oncogenesis of germinal center B cells (centroblasts) derived from naive B cells, from which the cells differentiate for the activation of genetic programs controlling DNA metabolism and pro-apoptotic programs and for the repression of anti-apoptotic pathways [36]. *FOXM1* is targeted by miR-1260 and miR-320, which were downregulated in our analysis, suggesting abnormal (increased) FOXM1 expression in DLBCL. Furthermore, in the negative regulation network FOXM1 also activates aurora kinase B (AURKB) which is part of a complex that is a key regulator of mitosis [37, 38]. Consequently, increased expression of activated AURKB could lead to enhancement of mitotic events and uncontrolled tumor growth. Interestingly, overexpression of AURKB is similarly regulated by miRs that have low expression levels in DLBCL (miR-let-7d and miR-let-7e). Our results suggest that polymerase II transcriptional factors are regulated by a large group of miRs, some of which play an important role in both positive and negative regulation of this pathway.

HHEX, a hematopoietically-expressed homeobox protein that is a transcriptional repressor with a role in hematopoietic differentiation [39, 40] is involved in both positive and negative regulation of the RNA Polymerase II promoter. Loss of HHEX in mice resulted in progressive loss of B lymphocytes in the circulation [39]. This was accompanied by the complete loss of B-cell progenitors in the bone marrow and loss of transitional B-cell subsets in the spleen. Xu et. al. have shown that HHEX is downregulated by miR-145-5p, suggesting that it could disrupt the differentiation process and consequently increase the probability of DLBCL development [41].

### Interaction networks in FL

**Figure 5:**
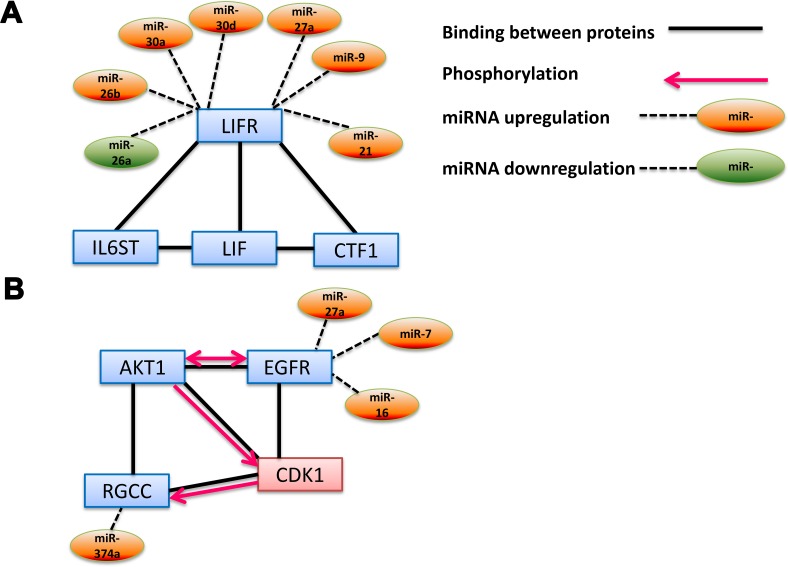
Interaction network pathways of miRs-mRNAs involved in FL **(A)** Leukemia inhibitory factor signaling pathway. The gene group (GO:0048861) was taken from Gene Ontology annotations analysis and related miRs from our screening. **(B)** Positive Regulation of Cyclin-dependent Protein Serine/threonine Kinase Activity Involved in G1/S Transition of Mitotic Cell Cycle. The gene group (GO:0031659) was taken from Gene Ontology annotations analysis. Up- and down regulated miRs were added to the network based on our screening. The charts are based on the STRING interaction database using all genes in these GO biological processes.

Unlike DLBCL, we did not find large control networks for FL (Figure 3B and Supplementary Table 3B). Nevertheless, we found a number of interesting connections that have a potential to be key factors in the development of the disease. One of the most prominent pathways found was the “Leukemia inhibitory factor signaling pathway” (GO:0048861) (Figure 3B), which is regulated by six overexpressed miRs (miRs-9, 21, 26b, 27a, 30a and 30d) and one downregulated miR (miR-26a) (Figure 5A). One of these miRs’ target belongs to leukemia inhibitory factor receptor (LIFR), which is a member of the cytokine receptor family that affects hematopoiesis as well as differentiation, survival, and proliferation of a wide variety of cells in adults and embryos. LIFR binds three additional proteins in this pathway: Leukemia inhibitory factor (LIF), interleukin 6 signal transduce (IC6GT) and cardiotrophin1 (CTF1) [42–44] (Figure 5A). LIF binds LIFR on the surface of the target cell, leading to downstream cellular processes such as transcription. Recently, LIFR was found to be associated with tumor maintenance by the MYC oncogene [45]. We hypothesize that this gene, whose expression is regulated by seven of our selected miRs, may be downregulated in FL similar to what was shown by Piccaluga [24], hence playing an important role in development of the disease.

Another interesting biological process detected by our GO analysis (highest score, 14.73, Supplementary Table 3B) is “Positive Regulation of Cyclin-dependent Protein Serine/threonine Kinase Activity Involved in G1/S Transition of Mitotic Cell Cycle” (GO:0031659). Our analysis revealed that miRs-374a, 27a, 7 and 16 regulate two genes that are involved in this pathway: regulator of cell cycle (RGCC) and endothelial growth factor receptor (EGFR). These two factors, together with the murine thymoma viral oncogene homolog (AKT1) bind cyclin-dependent kinase 1 (CDK1). Moreover, phosphorylation-dependent activity was also found between the proteins in the complex [46–50]. This complex plays an important role in cell cycle control, whereas its deregulation could accelerate tumor growth [51].

### Potential biomarkers of DLBCL

**Figure 6:**
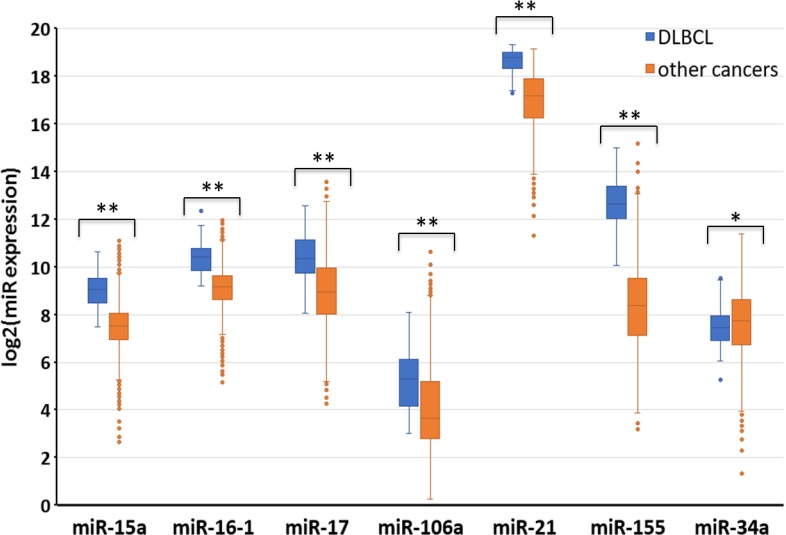
Distinct miR expression in DLBCL as compared to other types of malignancies The expression levels of seven miRs were compared between DLBCL and 31 other tumor types. The data was downloaded from the Cancer Genome Atlas through the UCSC XENA hubs [61]. The graph represents the expression of miRs in log2(RPM+1) values across 9405 samples (47 DLBCL and 9358 other tumor types). RPM = Read per million base pairs, ^**^p<0.01, ^***^p<0.0001 using Welch's *t*-test.

One of our goals was to identify groups of miRs that could be used as universal biomarkers for DLBCL and FL. We found a large group of unique miRs aberrantly expressed in DLBCL that are completely distinct from those expressed in FL (Figure 2), and chose seven unique miRs (miRs-15a, 16, 17-5p, 106, 21, 155 and 34a-5p) based on their overexpression in major bio-sources and their involvement in most of the biological processes that we analyzed in DLBCL (Figure 3A). Because these miRs have been suggested as potential biomarkers in several other cancer types [14, 16, 17], for example, miR-21 or miR-155 may be used as biomarkers for lung, brain, colon, prostate and bladder cancer, one miR alone cannot be used as a biomarker candidate for DLBCL diagnosis. We therefore compared the expression level of the selected miRs with the expression level of the same miRs in other cancers from the Pan-Cancer project (A total of 9405 patient samples). Because no relevant miR expression data were available on other hematological malignancies, we compared their expression levels to those of other malignancies. As shown in Figure 6, the expression levels of these miRs were statistically significantly higher in DLBCL compared with 31 other types of tumors (p<0.01 for miR-34a, p<0.0001 for miRs-155, 15a, 106a 16-1, 17 and 21). Thus, the specific expression profile of these miRs may be used as a possible diagnostic tool for DLBCL. Future studies are necessary in order to validate this miR signature in DLBCL compare to other hematological malignancies.

## MATERIALS AND METHODS

### Data sources and searches

We searched the online database, Medline, for scientific articles published between 2007 and 2017 that reported experimentally validated miR expression data from various human biological sources (tumor tissue, peripheral blood, bone marrow, and cell lines) and information on aberrantly regulated miRs in patients with DLBCL or FL as compared to healthy individuals. Articles were identified by the following key words in “All fields”: (1) DLBCL or FL; (2) microRNA or miRNA.

### Study selection

First, we screened all retrieved titles and abstracts for further review by verifying that the study contained original data and was relevant to the research question. From the initial title and abstract screening, articles were identified for full text review if the study was in humans, focused on DLBCL or FL malignancy, used healthy individuals as controls, and measured the expression of miRs in cells. We excluded from further analysis any letters, abstracts, editorials, and reviews, articles that were not in English, duplicate publications, articles containing information on prognosis and/or describing treatment of DLBCL or FL, and articles describing studies of lymphomas that included patients with the Epstein Barr virus (EBV) without comparisons to healthy individuals because DLBCL is rarely EBV-related [52, 53].

### Data extraction

From the selected publications we extracted the following information: authors, publication year, country, disease, miRs whose expression showed significant changes (up or downregulation) and their biological source. In the case of high throughput screening analysis, we collected only the information for 20 miRs, whose expression level was the most extensively altered in DLBCL or FL patients as compared to healthy individuals. Note that some of the aberrantly expressed miRs were reported to be upregulated in some studies, while in other studies the same miRs were downregulated, or vice versa. We did not classify such controversial miRs as “upregulated” or “downregulated”. As a result, the total number of up- and downregulated miRs is different than the total number of aberrantly expressed miRs that we found in the literature.

### Microarray gene analysis

The microarray dataset of purified miRs from tissue samples of DLBCL, FL and healthy individuals published by Piccaluga et al [24] was downloaded from the Gene Expression Omnibus database (http://www.ncbi.nlm.nih.gov/geo/, accession number GSE12195). A total of 131 samples were included in the dataset: 73 DLBCL, 38 FL and 20 controls. Gene expression data of all samples were preprocessed via background correction, quantile normalization and probe summarization using the Robust Multi-Array Average (RMA) algorithm in Affy software package of Bioconductor [54]. Probe annotation was performed using the annotate Bioconductor package [55]. Linear Models for Microarray Data (LIMMA) package [56] of Bioconductor was used to identify genes that were differentially expressed between the three groups DLBCL, FL and control. Only genes having adjusted p-value <0.05 and fold change >2 were chosen as differentially expressed and included in the downstream analysis.

### Ingenuity pathways analysis (IPA)

IPA (Ingenuity System Inc, USA, http://www.ingenuity.com) enables finding interactions between miRs and their mRNA targets. Using the “Core Analysis’ function included in IPA, we built a graphical representation of the molecular relationships between mRNAs whose expression significantly changed in DLBCL or FL compared to healthy controls. The sources of the validated miR data were TarBase, miRecords, and the peer-reviewed biomedical literature, as well as predicted miR-mRNA interactions from TargetScan [57]. The selected interactions between miRs and mRNAs were those with high confidence (predicted) or those with confirmed experimental observations.

### Gene ontology enrichment analysis

Gene ontology (GO) analysis of the genes detected by IPA as miR targets was done using Gene Analytics [58]. GO terms (e.g., super-pathways and GO-biological function) were given a score calculated by the software that was based on transformation of the binomial p-value, which is equivalent to a corrected p-value, with a significance defined at p <0.05.

### Network analysis

We used the STRING software [59] (version 10.0) to establish an interaction network between miRs and their targets in two high-scored GO biological processes: GO:0045944 and GO:0000122. The input consisted of two lists of proteins related to mentioned GO biological processes, 31 for GO:0045944 and 25 for GO:0000122 (Supplementary Table 3). To create the networks, we used the “enter multiple proteins” option, and chose “Homo-sapiens” as the organism. Proteins were selected based on their relation to “molecular action”. Next, the exiting target networks were combined with the miRs from the articles retrieved in the literature search and the direction of regulation (up- or downregulated) was determined.

### Data presentation

Pivot-tables were created using data paring between miRs and their targets retrieved by IPA. Each pair was marked by the biological source of its miR. Then, a script was written which automatically searched for all GO annotations related to the gene target of each miR-mRNA pair. This formed a new match between the GO index and the miRs. The data were then organized in pivot tables that marked the miRs by their biological source.

### Analysis of miR expression patterns using the UCSC XENA cancer genome browser

Expression levels of selected miRs in DLBCL were compared with expression levels of miRs defined in other common cancers. The analysis was performed using the UCSC XENA cancer genome browser [60] based on the Cancer Genome Atlas (TCGA) Pan-Cancer Cohort. A total of 9405 patient samples were used. MiR expression profiles as detected by miR-seq experiments in different tumors were visualized.

## CONCLUSIONS

In this work, we collected and analyzed data published between 2007 and 2017 on aberrantly expressed miRs from different biological sources in FL and DLBCL patients and in human cell lines. Based on these data, distinct patterns of abnormally expressed miRs were found for each disease, implying different molecular mechanisms.

Integrative analysis of abnormal miR and mRNA expression showed that each of these lymphoma subtypes has a unique set of miRs suggesting that distinct pathways are disrupted in FL and DLBCL.

To understand the functional associations between miRs, genes and processes that they regulate in DLBCL, we built an interaction network of the genes related to biological processes of “positive and negative regulation of transcription from RNA Polymerase II promoter”. Our results showed that each gene may be regulated by one or more miRs. Moreover, one miR can regulate a group of genes with related functions. Our results suggest that RNA polymerase II transcriptional factors are regulated by a large group of miRs, where some of them play an important role in both positive and negative regulation of this process.

Based on this analysis we chose seven miRs (miR-15a, 16, 17, 106, 21, 155 and miR-34a-5p) whose significant higher expression is specific to DLBCL then observed in other malignancies, suggesting that these miRs may be used as potential candidates’ biomarker for DLBCL diagnosis.

We were not able to analyze miRs by stages and subtypes, since there is not enough evidence to provide a statistically significant analysis yet. In order to provide clinical validity for this analysis future studies are needed. Moreover, only a few studies analyzed miR profiles in blood of DLBCL patients and we did not find any studies that examined miR signatures in the blood of FL patients.

The standard diagnostic protocol of many cancer types, including lymphoma, entails a histopathological inspection of tumor material obtained by invasive biopsy. Such a procedure is often expensive, uncomfortable and sometimes risky for patients. Therefore, we believe that, in the near future, circulating miRs from bio-fluids may be used as non-invasive cancer biomarkers. Moreover, miRs are much more stable than other RNA types such as long non-coding RNAs and mRNAs [61]. Hence, miRs may increase the ability to perform screening and repeat sampling on patients undergoing therapy or monitor disease progression, and allow for the development of a personalized approach to cancer patient treatment.

Further studies are required in order to explore possible circulating miRs as potential universal biomarkers for DLBCL and FL.

## SUPPLEMENTARY MATERIALS TABLES








