# Supplementary material for: Network analysis of microRNAs, genes and their regulation in diffuse and follicular B-cell lymphomas
Source: Oncotarget. 2018 Jan 5;9(8):7928–41. doi: 10.18632/oncotarget.23974 (PMC5814270; doi:10.18632/oncotarget.23974)
Supplement: Supplementary file 1 [file oncotarget-09-7928-s001.pdf]

Network analysis of microRNAs, genes and their regulation in diffuse and follicular B-cell lymphomas

SUPPLEMENTARY MATERIALS

Supplementary Table 1: Aberrantly expressed miRs in diffuse large B-cell lymphoma patients.

See Supplementary File 1

Supplementary Table 2: Aberrantly expressed miRs in Follicular lymphoma patients.

See Supplementary File 1

Supplementary Table 3: Aberrantly expressed miRs and genes in DLBCL were paired using Ingenuity Pathway Analysis. Pairs were labeled by colors according to their biological source. **(A)** 56 unique miRs and 845 genes in DLBCL generated 970 pairs. **(B)** 84 unique miRNAs and 60 genes in FL generated 90 pairs.

See Supplementary File 3

Supplementary Table 4: Target genes of DLBCL and FL were classified by functional groups using Gene Ontology (GO) analysis. DLBCL and FL related genes underwent function-based analysis using Gene Analytics software. The results include only high and medium score matches ($P<0.01$, $P<0.05$). **(A)** For DLBCL, 114 genes were included in the GO enrich results. **(B)** For FL, 13 genes were found.

See Supplementary File 4
