# Supplementary material for: Network analysis of microRNAs, genes and their regulation in diffuse and follicular B-cell lymphomas
Source: Oncotarget. 2018 Jan 5;9(8):7928–41. doi: 10.18632/oncotarget.23974 (PMC5814270; doi:10.18632/oncotarget.23974)
Supplement: Supplementary file 2 [file oncotarget-09-7928-s002.docx]

**Supplementary Tables:**

**Supplementary Table 1:** Aberrantly expressed miRs in diffuse large B-cell lymphoma patients.

| **Souse** | **Signatures** | **Expression** | **Comments/Reference** |
| --- | --- | --- | --- |
| Tumor Tissue | miR-210, miR-155, miR-106a, miR-17-5p | UP | Significantly higher expression in DBLCL than health control [1] |
| Tumor Tissue | miR-21 | UP | OncomiR overexpressed in most tumor types [2] |
| Tumor Tissue | miR-17-92 cluster (miR-17~92, miR-106a~363, and miR-106b~25) | UP | (miR-17-5p, miR-19b):  mainly in GC subtype [1], [3] |
| Tumor Tissue | miR-494, miR-638, miR-21, miR-155 | UP | Validated by two methods: (microarray and RT-qPCR) DLBCLs vs. LNs [4] |
| Tumor Tissue (DLBCL centroblast) | miR-10b-5p, miR-126-3p, miR-145-5p, miR-143-3p, miR-199a-5p, miR-99b-5p | UP | Six most abberantly expressed miRs when comparing DLBCL to Centroblast [5] |
| Tumor Tissue (DLBCL centroblast) | miR-10393-3p | UP | Novel miRs, more abundant in DLBCL patient samples than in centroblasts [5] |
| Tumor Tissue (DLBCL centroblast) | miR-10397-5p NOVELM00288M | UP | Novel miRs more abundant in ABC-DLBCL [5] |
| Tumor Tissue | miR-18b, miR-19b, miR-20a, miR-92a, miR-93, -miR-106a | UP | GC-DLBCL vs non-tumor lymph-nodes [6] |
| Cell lines | miR-17-92 cluster (miR-17~92, miR-106a~363, and miR-106b~25) | UP | miR-17-5p, miR-19b  mainly in GC subtype [2], [3] |
| Cell lines | miR-21 | UP | Onco-miR overexpressed in most tumor types [2] |
| Cell lines | miR-16, miR-20a, miR-29a, miR-106a | UP | ABC vs control [7] |
| Cell lines | miR-155, miR-21, miR-221, miR-222,  miR-518a, miR-363 | UP | ABC vs control[8] |
| Cell lines | miR-421, miR-324,  miR-590, miR-181a | UP | GCB vs control[8] |
| Blood Serum | miR-15, miR-210 and miR-21 | UP | Rechecked deliberately comparing levels of tumor-associated [9], [10] |
| Blood Serum | miR-15a, miR-16-1 and miR-29c | UP | [10] |
| Tumor Tissue | miR-150, miR-145, miR-328, miR-139, miR-95,  miR-99a, miR-10a, miR-149, miR-320, miR-151,  let-7e | DR/  lost | Significantly lower expression in DBLCL than health control [1] |
|  | miR-17-3, miR-595, miR-663 |  | most significantly lost in DBLCL [11], [12] |
| Tumor Tissue | miR-15a | DR | [13] |
| Tumor Tissue, Cell lines | miR-34a | DR | [14], [15] |
| Tumor Tissue | miR-150, miR-29b, miR-29a, miR-142-3p, miR-142-5p, miR-145, miR-143, miR-195, miR-497 | DR | Validated by two methods: (microarray and RT-qPCR) DLBCLs vs. LNs [4] |
| Cell lines | miR-17, miR-26a, let-7 (7d, 7g, 7i) | DR | ABC vs control [7] |
| Cell lines | miR-16, miR-19B, miR-26a, miR-29a, miR-720, miR-1260, miR-1280, let-7 (7D, 7G, 71) | DR | GCB vs control [7] |
| Cell lines | miR-26a, , let-7 (7D, 7G, 71) | DR | DLBCL vs control [7] |
| Cell lines | miR-17, miR-26, let-7 (7D, 7G, 7I) | DR | ABC vs control [7] |
| Blood Serum | 34a | DR | [10] |

**Supplementary Table 2:** Aberrantly expressed miRs in Follicular lymphoma patients

| **Souse** | **Signatures** | **Expression** | **Comments/Ref.** |
| --- | --- | --- | --- |
| Tumor Tissue | miR-9, miR-301, miR-213, miR-9*, miR-330,  miR-106a, miR-338, miR-155, miR-210 | UP | Significantly higher expression in FL than health control [1] |
| Tumor Tissue | miR-193a-5p, 193b*, -345, -513b, -574-3p, -584, -663, -1287, -1295, and -1471 | UP | Follicular lymphoma vs follicular hyperplasia [16] |
| Cell lines | miR-21 | UP | OncomiR overexpressed in most tumor types [2], [17] |
| Cell lines | miR-16, miR-19a, miR-19b,miR-20a, miR-20b, mir-92a, miR-92B, miR-106a, mir-487 | UP | FL vs control [7] |
| Bone Marrow | miR-338-5p, miR-200a, miR-24-2, miR-23a,miR-31, miR-639, miR-27a, miR-29a, miR-146b-3p,miR-374a, miR-766, miR-1271, miR-181a-2, miR-616, miR-7, 16 miR-30d, miR-885-5p, miR-941, miR-1208, miR-26b. | UP | Twenty most aberrantly expressed miRs in patients with FL, compared with healthy individuals (5 Patients) [18] |
| Tumor Tissue | miR-320, miR-149, miR-139 | DR | Significantly lower expression in FL than health control [1] |
| Tumor Tissue, Mouse Tumor Tissue | miR-34a | DR | [14], [15] |
| Tumor Tissue | miR-17*, -30a, -33a, -106a*, -141, -202, -205, -222, -301b, -431*, and -570 | DR | Follicular lymphoma vs follicular hyperplasia [16] |
| Tumor Tissue | miR-150 | DR | [6] |
| Cell lines | miR-34a | DR | [15] |
| Cell lines | miR-17, miR-26a, miR-29a, , let-7 (7D, 7G, 71) | DR | FL vs control [7] |
| Bone Marrow | miR-451, miR-144, miR-452, miR-494, miR-224, miR-486-3p, miR-483-3p,miR-190a, miR-10b,miR-939, miR-1248,miR-302b, miR-1303, miR-486-5p,miR-144, miR-511, miR-203a, miR-202, miR-204, miR-182 | DR | Twenty most aberrantly expressed miRs in patients with FL, compared with healthy individuals (5 Patients) [18] |

**References for Supplementary Table 1 and 2.**

1. Roehle A, Hoefig KP, Repsilber D, Thorns C, Ziepert M, Wesche KO, Thiere M, Loeffler M, Klapper W, Pfreundschuh M, Matolcsy A, Bernd HW, Reiniger L, et al. MicroRNA signatures characterize diffuse large B-cell lymphomas and follicular lymphomas. Br J Haematol. 2008; 142:732-44.

2. Lawrie CH, Soneji S, Marafioti T, Cooper CD, Palazzo S, Paterson JC, Cattan H, Enver T, Mager R, Boultwood J, Wainscoat JS, Hatton CS. MicroRNA expression distinguishes between germinal center B cell-like and activated B cell-like subtypes of diffuse large B cell lymphoma. Int J Cancer. 2007; 121:1156-61.

3. Lenz G, Wright GW, Emre NC, Kohlhammer H, Dave SS, Davis RE, Carty S, Lam LT, Shaffer AL, Xiao W, Powell J, Rosenwald A, Ott G, et al. Molecular subtypes of diffuse large B-cell lymphoma arise by distinct genetic pathways. Proc Natl Acad Sci U S A. 2008; 105:13520-5.

4. Mazan-Mamczarz K, Gartenhaus RB. Role of microRNAs and microRNA machinery in the pathogenesis of diffuse large B cell lymphoma. Leuk Res. 2013; 37:1420-8.

5. Lim EL, Trinh DL, Scott DW, Chu A, Krzywinski M, Zhao Y, Robertson AG, Mungall AJ, Schein J, Boyle M, Mottok A, Ennishi D, Johnson NA, et al.Comprehensive miRNA sequence analysis reveals survival differences in diffuse large B-cell lymphoma patients. Genome Biol. 2015; 16:18.

6. Fassina A, Marino F, Siri M, Zambello R, Ventura L, Fassan M, Simonato F, Cappellesso R. The miR-17-92 microRNA cluster: a novel diagnostic tool in large B-cell malignancies. Lab Invest. 2012; 92:1574-82.

7. Culpin RE, Proctor SJ, Angus B, Crosier S, Anderson JJ, Mainou-Fowler T. A 9 series microRNA signature differentiates between germinal centre and activated B-cell-like diffuse large B-cell lymphoma cell lines. Ann Intern Med. 2009; 151:414-20.

8. Lawrie CH, Saunders NJ, Soneji S, Palazzo S, Dunlop HM, Cooper CD, Brown PJ, Troussard X, Mossafa H, Enver T, Pezzella F, Boultwood J, Wainscoat JS, Hatton CS. MicroRNA expression in lymphocyte development and malignancy. Leukemia. 2008; 22:1440-6.

9. Lawrie CH, Gal S, Dunlop HM, Pushkaran B, Liggins AP, Pulford K, Banham AH, Pezzella F, Boultwood J, Wainscoat JS, Hatton CS, Harris AL. Detection of elevated levels of tumour-associated microRNAs in serum of patients with diffuse large B-cell lymphoma. Br J Haematol. 2008; 141:672-5.

10. Fang C, Zhu DX, Dong HJ, Zhou ZJ, Wang YH, Liu L, Fan L, Miao KR, Liu P, Xu W, Li JY. Serum microRNAs are promising novel biomarkers for diffuse large B cell lymphoma. Ann Hematol. 2012; 91:553-9.

11. Di Lisio L, Sánchez-Beato M, Gómez-López G, Rodríguez ME, Montes-Moreno S, Mollejo M, Menárguez J, Martínez MA, Alves FJ, Pisano DG, Piris MA, Martínez N. MicroRNA signatures in B-cell lymphomas. Blood Cancer J. 2012; 2:e57.

12. Lawrie CH, Chi J, Taylor S, Tramonti D, Ballabio E, Palazzo S, Saunders NJ, Pezzella F, Boultwood J, Wainscoat JS, Hatton CS. Expression of microRNAs in diffuse large B cell lymphoma is associated with immunophenotype, survival and transformation from follicular lymphoma. J Cell Mol Med. 2009; 13:1248-60.

13. Eis PS, Tam W, Sun L, Chadburn A, Li Z, Gomez MF, Lund E, Dahlberg JE. Accumulation of miR-155 and BIC RNA in human B cell lymphomas. Proc Natl Acad Sci U S A. 2005; 102:3627-32.

14. He M, Gao L, Zhang S, Tao L, Wang J, Yang J, Zhu M. Prognostic significance of miR-34a and its target proteins of FOXP1, p53, and BCL2 in gastric MALT lymphoma and DLBCL. Gastric Cancer. 2014; 17:431-41.

15. Craig VJ, Cogliatti SB, Imig J, Renner C, Neuenschwander S, Rehrauer H, Schlapbach R, Dirnhofer S, Tzankov A, Müller A. Myc-mediated repression of microRNA-34a promotes high-grade transformation of B-cell lymphoma by dysregulation of FoxP1. Blood. 2011; 117:6227-36.

16. Wang W, Corrigan-Cummins M, Hudson J, Maric I, Simakova O, Neelapu SS, Kwak LW, Janik JE, Gause B, Jaffe ES, Calvo KR. MicroRNA profiling of follicular lymphoma identifies microRNAs related to cell proliferation and tumor response. Haematologica. 2012; 97:586-94.

17. Yamanaka Y, Tagawa H, Takahashi N, Watanabe A, Guo YM, Iwamoto K, Yamashita J, Saitoh H, Kameoka Y, Shimizu N, Ichinohasama R, Sawada KI. Aberrant overexpression of microRNAs activate AKT signaling via down-regulation of tumor suppressors in natural killer-cell lymphoma/leukemia. Blood. 2009; 114:3265-75.

18. Takei Y, Ohnishi N, Kisaka M, Mihara K. Determination of abnormally expressed microRNAs in bone marrow smears from patients with follicular lymphomas. Springerplus. 2014; 3:1-9.
